# Computed tomography perfusion imaging-guided intravenous thrombolysis in acute minor ischemic stroke

**DOI:** 10.3389/fneur.2023.1284058

**Published:** 2023-11-27

**Authors:** Jennifer Sartor-Pfeiffer, Mirjam Lingel, Maria-Ioanna Stefanou, Markus Krumbholz, Florian Hennersdorf, Ulrike Ernemann, Sven Poli, Katharina Feil, Ulf Ziemann, Annerose Mengel

**Affiliations:** ^1^Department of Neurology and Stroke, University of Tübingen, Tübingen, Germany; ^2^Hertie-Institute for Clinical Brain Research, University of Tübingen, Tübingen, Germany; ^3^Department of Neurology and Pain Treatment, MS Center, Center for Translational Medicine, Immanuel Klinik Rüdersdorf, University Hospital of the Brandenburg Medical School Theodor Fontane, Rüdersdorf bei Berlin, Germany; ^4^Faculty of Health Sciences Brandenburg, Brandenburg Medical School Theodor Fontane, Rüdersdorf bei Berlin, Germany; ^5^Department of Diagnostic and Interventional Neuroradiology, University of Tübingen, Tübingen, Germany

**Keywords:** minor stroke, ischemic stroke, computed tomography perfusion, intravenous thrombolysis, imaging

## Abstract

**Background:**

Over 50% of acute ischemic stroke (AIS) patients present with minor neurological deficits, and optimal treatment is still debated. The randomized PRISMS trial did not show beneficial effects of intravenous thrombolysis (IVT) in unselected patients with minor stroke and non-disabling neurological deficits.

**Purpose:**

The study aimed to evaluate if AIS patients with minor stroke may benefit from computed-tomography-perfusion (CTP)-guided IVT. The primary endpoint was good functional outcomes, defined as a modified Rankin Scale score of 0–2 at 90 days.

**Methods:**

AIS patients with a NIHSS of ≤5 presenting within 4.5 h underwent multimodal CT-imaging including CTP. CTP mismatch was defined as hypoperfusion on CTP with time-to-peak delay >6 s without corresponding hypoperfusion in cerebral blood volume. IVT decision was left to the attending stroke physicians. Patients with large vessel occlusion (LVO) and absolute contraindications to IVT were excluded.

**Results:**

In total, 267 consecutive patients were included [mean age: 72 ± 14 years, 45.3% female patients, 75.3% received IVT, median NIHSS on admission: 3 (IQR 2, 4)]. CTP mismatch was detected in 41.8% of IVT− treated patients (IVT+) and 28.8% of standard treatment patients (IVT−) (*p* = 0.06). IVT+ had favorable outcomes at 90 days compared to IVT− (*p* = 0.006), but no interaction with an existing CTP mismatch was detected (OR_adj_: 1.676; 95% CI: 0.644–4.364). No symptomatic intracranial hemorrhage according to ECASS-III criteria occurred.

**Conclusion:**

Although selected AIS patients with minor stroke may benefit from IVT, CTP mismatch does not correlate with functional outcomes. No benefit from CTP mismatch in guiding IVT was detected in patients without LVO presenting with minor neurological deficits.

## 1 Introduction

Intravenous thrombolysis (IVT) within 4.5 h after stroke symptom onset is the standard of care in patients with disabling acute ischemic stroke (AIS) (1–3). Patients with minor neurological deficits [National Institutes of Health Stroke Scale (NIHSS) score ≤5] represent more than 50% of all AIS patients (4, 5). The optimal treatment for these patients is still a matter of debate and practices vary across stroke centers and countries (6). The randomized PRISMS trial did not show beneficial effects of intravenous thrombolysis (IVT) in unselected patients with minor, not clearly disabling neurological deficits (5). However, early neurological deterioration is not uncommon in minor stroke and is associated with disabling functional outcomes (7, 8). In an analysis of patients who did not receive IVT because of minor stroke or rapidly improving symptoms, nearly 25% had an unfavorable outcome (an mRS of 2–6) (9) or could not be discharged directly home (10). Hence, the question of how to select minor stroke patients who may benefit from IVT still remains unanswered.

Multimodal computed tomography (CT) imaging with CT angiography (CTA) and CT perfusion (CTP) could facilitate the identification of patients who may benefit from IVT. For major strokes, the mismatch concept has been established in numerous clinical trials for the evaluation of the hypoperfused salvageable brain tissue (infarct penumbra) vs. irreversibly injured brain areas (infarct core) (11, 12). Moreover, in minor ischemic stroke, the analysis of a prospective registry identified the presence of CTP mismatch as the strongest predictor of disability at 90 days (13). A case series of 73 minor ischemic stroke patients (defined as NIHSS ≤3) with demonstrable penumbra on CTP imaging showed better functional outcomes at follow-up after IVT treatment compared to 39 standard care patients (14). To date, however, the value of perfusion imaging in acute minor ischemic stroke is not well established.

The aim of our study was to examine whether AIS patients with minor neurological deficits presenting within the therapeutic time window may benefit from CTP− guided IVT. Furthermore, we sought to investigate whether CTP mismatch may be a relevant prognosticator of clinical outcomes in minor stroke patients undergoing standard treatment.

## 2 Materials and methods

### 2.1 Study population

We performed a retrospective analysis of our prospective local stroke registry at the University Hospital Tübingen (EC number 189/2019BO2) between 01/2013 and 03/2019. The inclusion criteria were as follows: AIS patients admitted within 4.5 h after stroke onset, minor stroke severity (defined as NIHSS ≤5 points on admission), and multimodal CT imaging [non-contrast CT (ncCT), CTA, and CTP] on admission. The exclusion criteria were as follows: large vessel occlusion (LVO) in CTA, absolute contraindications to IVT (i.e., therapeutic anticoagulation and metastasized neoplasm), and treatment with endovascular thrombectomy. IVT decision per case was left to the discretion of the attending stroke physician and administered as alteplase (Actilyse^®^) at a dosage of 0.9 mg/kg body weight in accordance with national (German Neurological Society) (1, 2, 15) and international guidelines [European Stroke Organization (3) and American Heart Association/American Stroke Association (16, 17)]. Off-label IVT was performed after written informed consent. The attending stroke physician decided about secondary prophylactic medication for patients. Standard doses in accordance with national guidelines were used (1, 15). Outcome variables included functional outcomes using the NIHSS at 24 h and the NIHSS difference between admission and 24 h and between admission and discharge. The degree of dependence or disability was rated by the modified Rankin scale (mRS). A good clinical outcome was defined as an mRS of 0–2 (18). Clinical follow-up data were acquired at 3 months via a structured telephone interview. Any intracranial hemorrhage (ICH) was defined as any symptomatic or asymptomatic ICH including hemorrhagic transformation in cerebral infarct. Symptomatic ICH (sICH) was defined according to ECASS-III (19).

### 2.2 Imaging data

Upon admission, each patient underwent CT imaging including ncCT, CTA, and CTP. In ncCT, the Alberta stroke program early CT score (ASPECTS) was determined visually by senior neuroradiologists and used to quantify infarct demarcation on admission. For CTA, 50 mL of the iodinated contrast agent was administered intravenously, followed by a saline chaser of 40 mL, both with a flow rate of 5 mL/s. CTA was performed from the aortic arch to the vertex with 140 and 80 kV tube voltage and attenuation-based tube current modulation (CareDose). Collimation was 0.6 mm. CTA data were read as source images using syngo.via imaging software (Siemens Healthcare, Erlangen, Germany). CTP was obtained with 0.6 mm collimation and 100 mm scan coverage in the *z*-axis using adaptive spiral scanning. The datasets were acquired continuously over 48 s (32 cycles, one sweep every 1.5 s). Tube voltage and current were 80 kV and 200 mAs, respectively. A total of 35 mL of iodinated contrast agent (400 mg/mL) was administered at a flow rate of 5 mL/s, followed by a saline flush of 40 mL at 5 mL/s. The analysis of CTP was based on the cerebral blood flow (CBF) and cerebral blood volume (CBV) perfusion maps. The CTP mismatch according to the ASPECTS topography was assessed visually based on CBF and CBV maps as previously described (20). In addition, the mismatch was visually assessed in 10% increments as routinely used clinically and as previously described (21). CTP mismatch was defined as a sign of hypoperfusion in CTP with increased time-to-peak without corresponding infarct core in CBV, whereby changes with a delay of >6 s were included (20). All images were independently assessed by two senior neuroradiologists, who were blinded for clinical data; any disagreements were settled by mutual consensus for the final acquisition of mismatch metrics.

### 2.3 Statistical analysis

Continuous variables are expressed as mean and standard deviation or median with interquartile range (IQR). Categorical data are presented as proportions. Group differences between baseline patient demographics and clinical characteristics were assessed using Pearson’s chi-squared test and the Wilcoxon–Mann–Whitney test depending on data characteristics (i.e., categorical vs. continuous), respectively. Comparison of the primary outcome was considered statistically significant for a two-sided *p*-value of <0.05. Bonferroni correction was applied to adjust for multiple testing of secondary outcomes. In the primary analysis, logistic regression analysis was performed to investigate whether CTP mismatch may be associated with good clinical outcomes at 90 days (defined as an mRS of 0–2) following IVT in minor ischemic stroke patients. In secondary sensitivity analyses, logistic regression analyses were performed to assess the prognostic yield of CTP in relation to clinical outcomes among the following patients: (i) those who underwent IVT and (ii) those who underwent standard treatment. Statistical analyses were performed using statistical software program IBM SPSS^®^ Statistics for Mac, version 26 (IBM Corp., Armonk, NY, United States) and JMP^®^ for Windows version 15.2 and 16.2.0 (SAS Institute Inc., Cary, NC, United States). We used binomial distribution and logit function to compare outcome variables. Results are reported in accordance with the STROBE guidelines (22). The figures presented were created with Microsoft PowerPoint.

## 3 Results

### 3.1 Primary analysis

**Figure 1:**
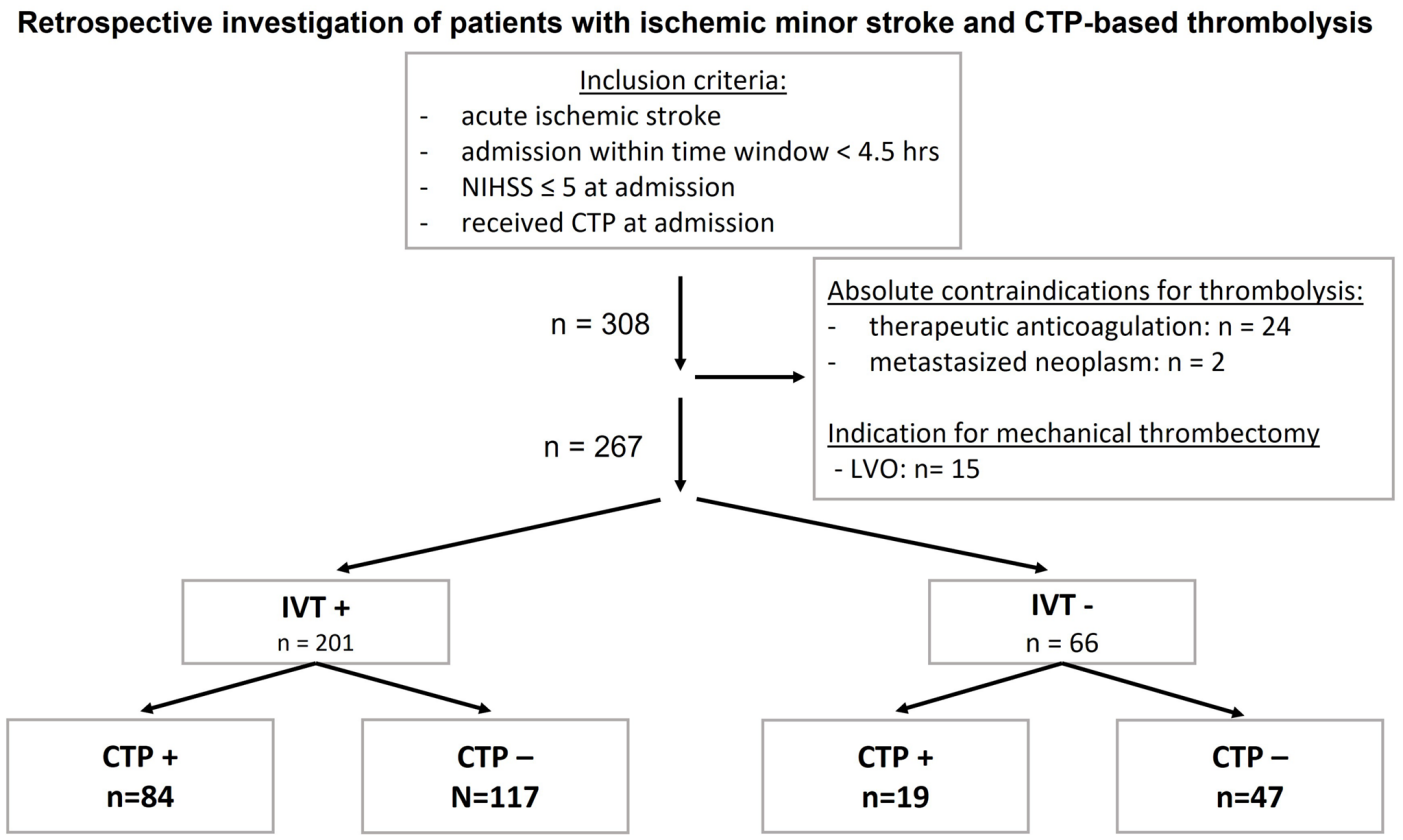
Inclusion criteria and group sizes of patients with acute ischemic minor stroke and CTP− guided thrombolysis. Patients with absolute contraindications for IVT were excluded. CTP, computed tomography perfusion; CTP−, CTP negative, no mismatch on CTP; CTP+, CTP positive, mismatch in CTP; hrs, hours; IVT, intravenous thrombolysis; IVT−, patients who did not receive IVT; IVT+, patients who received IVT; *n*, number, LVO, large vessel occlusion; NIHSS, National Institutes of Health Stroke Scale.

A total of 267 patients [mean age 72 ± 14 years, 45.3% female patients, median NIHSS on admission: 3; (IQR 2, 4)] were included, of whom 201 patients (75.3%) received IVT (denoted as IVT+) and 66 patients (24.7%) received the best medical treatment (denoted as IVT−) (Figure 1). On CTP imaging, 41.8% of IVT+ patients and 28.8% of standard treatment patients had CTP mismatch (*p* = 0.06) with a higher probability of IVT administration in patients with CTP mismatch (denoted as CTP+) compared to patients without CTP mismatch (denoted as CTP−).

Baseline demographic, clinical, and radiological data of IVT+ and IVT− are summarized in Table 1. There were no significant differences between groups regarding age and sex. There was a significant difference in ASPECTS on ncCT on admission (*p* = 0.03). IVT patients had significantly higher rates of prehospital neurological improvement (*p* < 0.001) compared to IVT+. In addition, IVT+ were found to have higher NHSS on admission (*p* < 0.001).

**Table 1:** Baseline characteristics of minor ischemic stroke patients with or without IVT.

	IVT+ (*n* = 201)	IVT− (*n* = 66)	*p*-value IVT+ vs. IVT−
Age, years (SD)	72 ± 14	73 ± 14	0.70
Sex, female^a^	90 (44.8)	31 (47)	0.76
**Medical history**			
Hypertension^a^	153 (76.1)	50 (75.8)	0.95
History of smoking^a^	21 (10.5)	14 (21.2)	**0.02**
Hyperlipidemia^a^	60 (29.9)	24 (36.4)	0.32
Diabetes^a^	47 (23.4)	16 (24.2)	0.89
Prior AIS/TIA^a^	67 (33.3)	12 (18.2)	**0.02**
Atrial fibrillation^a^	36 (17.9)	9 (13.6)	0.42
**Symptomatic**			
Paresis^a^	111 (55.2)	35 (53.0)	0.76
Hypesthesia^a^	42 (20.9)	14 (21.2)	0.96
Aphasia^a^	68 (33.8)	9 (13.6)	**0.002**
Neglect^a^	8 (4.0)	1 (1.5)	0.34
Dysarthria^a^	66 (32.8)	26 (39.4)	0.33
Dizziness^a^	36 (18.0)	7 (10.6)	0.16
**Brain imaging**			
ASPECTS^b^	10 (10, 10)	10 (10, 10)	**0.03**
**Qualifying event**			
Prehospital neurological improvement^a^	44 (21.9)	30 (47.6)	**<0.001**
Admission NIHSS^b^	4 (2, 4)	2 (1, 3)	**<0.001**
Onset-to-admission time (min)^b^	80 (55, 135)	110 (55, 151)	0.10
Onset-to-IVT time (min)^b^	120 (90, 180)	—	—
Door-to-needle time (min)^b^	34 (25, 45)	—	—

ASPECTS is calculated based on the results of CT on admission. Prehospital neurological improvement describes an improvement in neurological symptoms rated by NIHSS on admission compared to the severity of symptoms at the onset of symptoms. Significant results (*p* < 0.05) are in bold. ASPECTS, Alberta stroke program early CT score; CT, computed tomography; CTP, computed tomography perfusion; CTP−, CTP negative, no mismatch on CTP; CTP+, CTP positive, mismatch in CTP; IVT, intravenous thrombolysis; IVT−, patients who did not receive IVT; IVT+, patients who received IVT; min, minute; *n*, number; NIHSS, National Institutes of Health Stroke Scale; SD, standard deviation; vs., versus.

a Number (%), calculated with Pearson’s chi-squared test.

b Median (interquartile range), calculated with the Wilcoxon–Mann–Whitney test.

Comparing IVT+ vs. IVT−, significant differences in NIHSS after 24 h (*p* = 0.008) were disclosed (Table 2). IVT+ performed significantly better regarding the NIHSS difference between admission and 24 h (*p* < 0.001) and the NIHSS difference between admission and discharge (*p* < 0.001). At follow-up, significantly lower mRS at 90 days was disclosed for IVT+ (*p* = 0.006) compared to IVT−. Moreover, stratified analyses for an mRS of 0–1 after 90 days (*p* = 0.002) and an mRS of 0–2 after 90 days (*p* = 0.009) revealed similar results. Furthermore, the mRS difference between admission and discharge (*p* < 0.001) and the mRS difference between admission and 90 days (*p* < 0.001) differed significantly between groups.

**Table 2:** Outcomes of minor ischemic stroke patients with or without IVT.

	IVT+ (*n* = 201)	IVT− (*n* = 66)	*p*-value IVT+ vs. IVT−
NIHSS after 24 h^b^	1 (0, 3)	1 (0, 2)	**0.008**
NIHSS difference between admission—24 h^b^^,^^c^	−2 (−3, 0)	−1 (−2, 0)	**<0.001**
NIHSS difference between admission—discharge^b^^,^^c^	−3 (−4, −2)	−1 (−2, 0)	**<0.001**
mRS at discharge^b^	1 (0, 2)	1 (0, 2)	0.61
mRS difference between admission and discharge^b^^,^^c^	−1 (−2, 0)	0 (0, 1)	**<0.001**
mRS difference between admission and 90 days^b^^,^^c^	−2 (−3, −1)	0 (0, 1)	**<0.001**
mRS after 90 days^b^	0 (0, 1)	1 (0, 2)	**0.006**
mRS after 90 days 0–1^b^	0 (0, 0)	0 (0, 1)	**0.002**
mRS after 90 days 0–2^b^	0 (0, 1)	1 (0, 1)	**0.009**
Mortality after 90 days^a^	2 (1.1)	1 (1.6)	0.73
**Complications of IVT**			
sICH^a^	0 (0)	0 (0)	—
Any ICH^a^	9 (4.5)	0 (0)	0.08
**Stroke etiology according to TOAST classification**			
TOAST 1^a^	38 (18.9)	14 (21.2)	0.68
TOAST 2^a^	66 (32.8)	14 (21.2)	0.07
TOAST 3^a^	22 (11.0)	15 (22.7)	**0.02**
TOAST 4^a^	8 (4.0)	1 (1.5)	0.34
TOAST 5^a^	67 (33.3)	22 (33.3)	1.0

Significant results (*p* < 0.05) are in bold. Any ICH is defined as any symptomatic or asymptomatic ICH including hemorrhagic transformation in cerebral imaging. CTP, computed tomography perfusion; CTP−, CTP negative, no mismatch in CTP; CTP+, CTP positive, mismatch in CTP; d, days; ICH, intracerebral hemorrhage; IVT, intravenous thrombolysis; IVT−, patients who did not receive IVT; IVT+, patients who received IVT; min, minutes; mRS, modified Rankin Scale; *n*, number; NIHSS, National Institutes of Health Stroke Scale; sICH, symptomatic ICH according to ECASS-III criteria; TOAST, classification of subtypes of acute ischemic stroke due to “Trial of Org 10,172 in Acute Stroke Treatment”; vs., versus.

a Number (%).

b Median (IQR).

c Negative results imply a reduction of NIHSS or mRS in time course, positive results imply an increase of NIHSS or mRS in time course.

**Figure 2:**
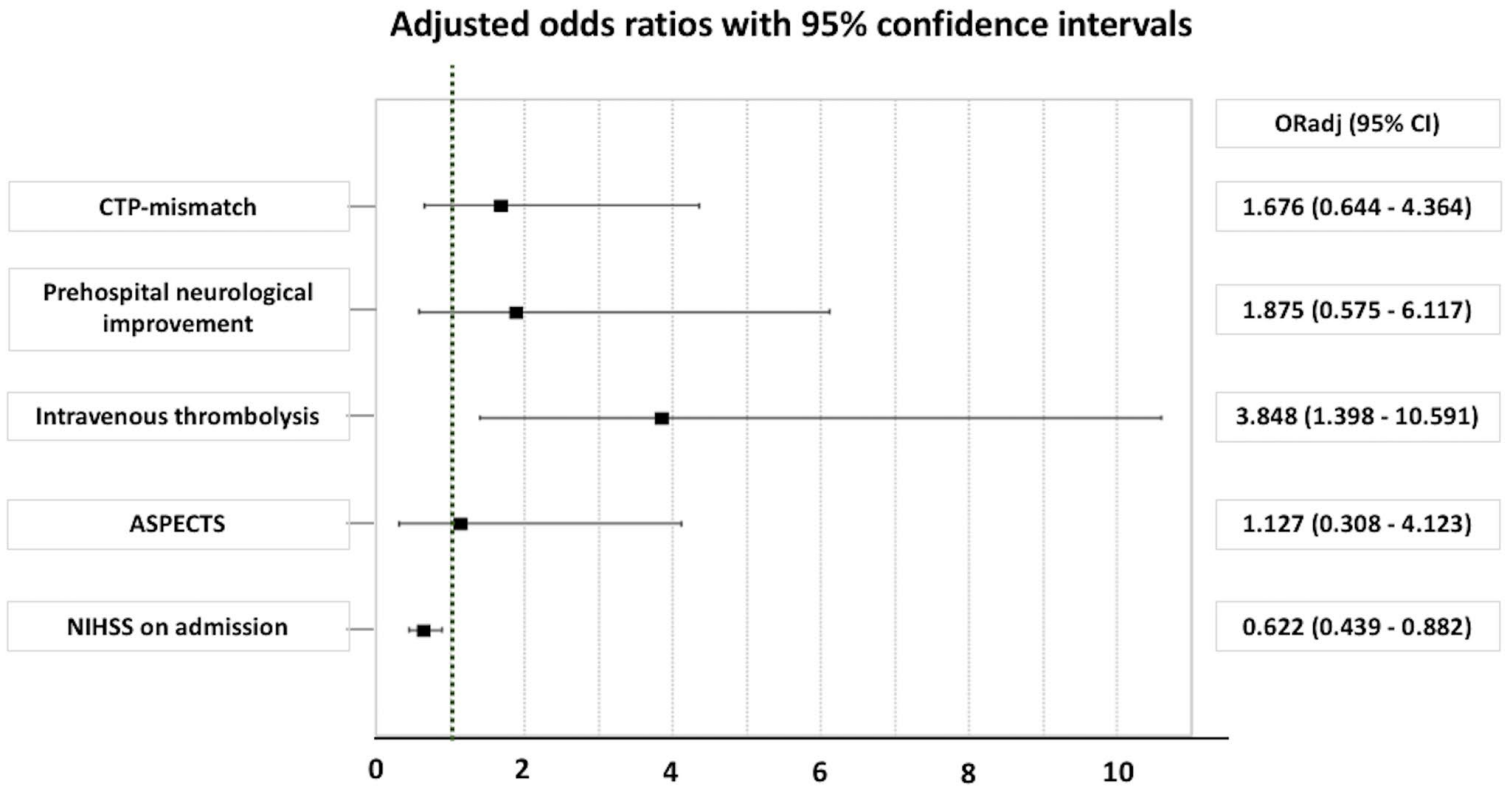
Adjusted odds ratio for good outcome (an mRS of 0–2) at 90 days in patients with minor ischemic stroke. ASPECTS, Alberta stroke program early computed tomography score; CI, confidence interval; CTP, computed tomography perfusion; NIHSS, National Institutes of Health Stroke Scale; OR_adj_, adjusted odds ratio.

In logistic regression analyses, after adjustment for baseline differences between IVT+ vs. IVT− patients (Figure 2), a significant association between IVT and good functional outcome at 90 days (defined as an mRS of 0–2) was disclosed (*p* = 0.009), whereas NIHSS on admission was negatively associated with good functional outcomes at 90 days (*p* = 0.008). However, no association between CTP mismatch and good functional outcomes at 90 days (an mRS of 0–2) was detected (*p* = 0.290).

No sICH according to the ECASS-III criteria occurred. Among patients receiving IVT, 9 patients (4.5%) suffered from any ICH, and no ICH occurred in the standard treatment group (*p* = 0.08) (Table 2).

### 3.2 Secondary sensitivity analyses

For further analysis, patients were divided into groups according to their treatment (IVT vs. standard treatment), and dichotomous analyses were performed based on the presence of CTP mismatch: (i) CTP + IVT+ (*n* = 84) vs. CTP − IVT+ (*n* = 117) and (ii) CTP + IVT– (*n* = 19) vs. CTP − IVT– (*n* = 47) (Table 3).

**Table 3:** Baseline characteristics of minor ischemic stroke patients with or without IVT and with or without CTP mismatch.

	CTP + IVT+ (*n* = 84)	CTP − IVT+ (*n* = 117)	CTP + IVT− (*n* = 19)	CTP − IVT− (*n* = 47)	*p*-value CTP + IVT+ vs. CTP − IVT+	*p*-value CTP + IVT− vs. CTP − IVT−
Age, years (SD)	72 (±13)	72 (±14)	73 (15)	73 (13)	0.83	0.90
Sex, female^a^	36 (42.9)	54 (46.2)	10 (52.6)	21 (44.7)	0.64	0.56
**Medical history**						
Hypertension^a^	58 (69.1)	95 (81.2)	12 (63.2)	38 (80.9)	**0.046**	0.13
History of smoking^a^	9 (10.7)	12 (10.3)	3 (15.8)	11 (23.4)	0.92	0.49
Hyperlipidemia^a^	28 (33.3)	32 (27.4)	11 (57.9)	13 (27.7)	0.36	**0.02**
Diabetes^a^	21 (25.0)	26 (22.2)	5 (26.3)	11 (23.4)	0.65	0.80
Prior AIS/TIA^a^	26 (31.0)	41 (35.0)	4 (21.1)	8 (17.0)	0.54	0.70
Atrial fibrillation^a^	18 (21.4)	18 (15.4)	4 (21.1)	5 (10.6)	0.27	0.26
**Symptomatic**						
Paresis^a^	42 (50.0)	69 (59.0)	9 (47.4)	26 (55.3)	0.21	0.56
Hypesthesia^a^	11 (13.1)	31 (26.5)	8 (42.1)	6 (12.8)	**0.02**	**0.008**
Aphasia^a^	33 (39.3)	35 (29.9)	7 (36.8)	2 (4.3)	0.17	**0.001**
Neglect^a^	5 (6.0)	3 (2.6)	1 (5.3)	0 (0)	0.23	0.11
Dysarthria^a^	23 (27.4)	43 (36.8)	5 (26.3)	21 (44.7)	0.16	0.17
Dizziness^a^	8 (9.5)	25 (21.4)	0 (0)	7 (14.9)	**0.03**	0.08
**Brain imaging**						
ASPECTS^b^	10 (10, 10)	10 (10, 10)	10 (10, 10)	10 (10, 10)	0.18	0.12
**Qualifying event**						
Prehospital neurological improvement^a^	28 (33.3)	16 (13.7)	13 (72.2)	17 (37.8)	**0.001**	**0.01**
Admission NIHSS^b^	3 (2, 4)	4 (3, 5)	2 (0, 3)	2 (1, 3)	0.09	0.59
Onset-to-admission time (min)^b^	64 (41, 100)	93 (60, 139)	78 (53, 150)	114 (59, 153)	**0.001**	0.67
Onset-to-IVT time (min)^b^	109 (79, 153)	130 (99, 185)	—	—	**0.004**	—
Door-to-needle time (min)^b^	35 (25, 45)	32 (24, 46)	—	—	0.68	—

ASPECTS is calculated based on results of CT on admission. Prehospital neurological improvement describes an improvement in neurological symptoms rated by NIHSS on admission compared to the severity of symptoms at the onset of symptoms. Significant results (*p* < 0.05) are in bold. ASPECTS, Alberta stroke program early CT score; CT, computed tomography; CTP, computed tomography perfusion; CTP−, CTP negative, no mismatch in CTP; CTP+, CTP positive, mismatch in CTP; IVT, intravenous thrombolysis; IVT−, patients who did not receive IVT; IVT+, patients who received IVT; min, minute; *n*, number; NIHSS, National Institutes of Health Stroke Scale; SD, standard deviation; vs., versus.

a Number (%), calculated with Pearson’s chi-squared test.

b Median (interquartile range), calculated with Wilcoxon–Mann–Whitney test.

#### 3.2.1 Prognostic yield of CTP mismatch on IVT outcomes in acute minor ischemic stroke patients (CTP + IVT+ vs. CTP − IVT+)

Among patients who underwent IVT, patients with CTP mismatch showed significantly more prehospital neurological improvement (*p* = 0.001) (Table 3). CTP + IVT+ showed significantly shorter onset-to-admission-time (*p* = 0.001) and shorter onset-to-IVT− time (*p* = 0.004) than CTP − IVT+. Door-to-needle time was similar between CTP + IVT+ and CTP − IVT+ (*p* = 0.68). NIHSS on admission did not differ between CTP + IVT+ vs. CTP − IVT+ (*p* = 0.09) and CTP + IVT− vs. CTP − IVT− (*p* = 0.59).

With respect to clinical parameters, groups differed to some extent in medical history and neurological symptoms on admission. CTP − IVT+ were found with a higher frequency of hypertension (81.2% vs. 69.1%, *p* = 0.046) and showed more often hypesthesia (26.5% vs. 13.1%, *p* = 0.02) and dizziness (21.4% vs. 9.5%, *p* = 0.03) compared to CTP + IVT+. Microangiopathic stroke etiology (TOAST 3) was significantly more frequent in CTP − IVT+ (17.1% vs. 2.4%, *p* = 0.001).

With respect to clinical outcomes, CTP + IVT+ and CTP − IVT+ did not differ regarding NIHSS after 24 h (*p* = 0.23). Both groups benefited from IVT as reflected by the NIHSS difference between admission and 24 h (*p* = 0.01) and the NIHSS difference between admission and discharge (*p* = 0.03). There was no significant difference in the mRS difference between admission and discharge (*p* = 0.89), in the mRS difference between admission and 90 days (*p* = 0.89), and in mRS after 90 days (*p* = 0.46) with overall low mRS after 90 days; similar results were also obtained in detailed analysis for an mRS of 0–1 after 90 days (*p* = 0.41) and an mRS of 0–2 after 90 days (*p* = 0.82). There was no significant difference in the frequency of any ICH (CTP + IVT+ 3.6% vs. CTP − IVT+ 5.1%, *p* = 0.60). No sICH according to ECASS-III criteria occurred (Table 4).

**Table 4:** Outcomes of minor ischemic stroke patients with or without IVT and with or without CTP mismatch.

	CTP + IVT+ (*n* = 84)	CTP − IVT+ (*n* = 117)	CTP + IVT− (*n* = 19)	CTP − IVT− (*n* = 47)	*p*-value CTP + IVT+ vs. CTP − IVT+	*p*-value CTP + IVT− vs. CTP − IVT−
NIHSS after 24 h^b^	1 (1, 3)	1 (0, 2)	0 (0, 1)	1 (0, 2)	0.23	0.34
NIHSS difference between admission—24 h^b^^,^^c^	−2 (−2, 0)	−2 (−3, −1)	0 (−2, 0)	−1 (−2, 0)	**0.01**	0.93
NIHSS difference between admission—discharge^b^^,^^c^	−2 (−3, −1)	−3 (−4, −2)	0 (−2, 0)	−1 (−2, 0)	**0.03**	0.97
mRS at discharge^b^	1 (0, 2)	1 (0, 2)	0 (0, 1)	1 (0, 2)	0.36	**0.002**
mRS difference between admission and discharge^b^^,^^c^	−1 (−2, 0)	−1 (−2, 0)	0 (0, 0)	0 (0, 1)	0.89	0.13
mRS difference between admission and 90 days^b^^,^^c^	−2 (−3, −1)	−2 (−3, −1)	0 (0, 0)	0 (0, 1)	0.89	0.87
mRS after 90 days^b^	0 (0, 1)	0 (0, 1)	0 (0, 1)	1 (0, 2)	0.46	**0.01**
mRS after 90 days 0–1^b^	0 (0, 1)	0 (0, 0)	0 (0, 0)	1 (0, 1)	0.41	**0.008**
mRS after 90 days 0–2^b^	0 (0, 1)	0 (0, 1)	0 (0, 1)	1 (0, 1)	0.82	**0.006**
Mortality after 90 days^a^	0 (0)	2 (1.8)	0 (0)	1 (2.3)	0.23	0.50
**Complications of IVT**						
sICH^a^	0 (0)	0 (0)	0 (0)	0 (0)	—	—
Any ICH^a^	3 (3.6)	6 (5.1)	0 (0)	0 (0)	0.60	—
**Stroke etiology according to TOAST classification**						
TOAST 1^a^	17 (20.2)	21 (18.0)	5 (26.3)	9 (19.2)	0.68	0.52
TOAST 2^a^	33 (39.3)	33 (28.2)	5 (26.3)	9 (19.2)	0.10	0.52
TOAST 3^a^	2 (2.4)	20 (17.1)	1 (5.3)	14 (29.8)	**0.001**	**0.03**
TOAST 4^a^	3 (3.6)	5 (4.3)	1 (5.3)	0 (0)	0.80	0.11
TOAST 5^a^	29 (34.5)	38 (32.5)	7 (36.8)	15 (31.9)	0.76	0.70

Significant results (*p* < 0.05) are in bold. Any ICH is defined as any symptomatic or asymptomatic ICH including hemorrhagic transformation in cerebral imaging. CTP, computed tomography perfusion; CTP−, CTP negative, no mismatch in CTP; CTP+, CTP positive, mismatch in CTP; d, days; ICH, intracerebral hemorrhage; IVT, intravenous thrombolysis; IVT−, patients who did not receive IVT; IVT+, patients who received IVT; min, minutes; mRS, modified Rankin Scale; *n*, number; NIHSS, National Institutes of Health Stroke Scale; sICH, symptomatic intracerebral hemorrhage according to ECASS-III criteria; TOAST, classification of subtypes of acute ischemic stroke due to “Trial of Org 10,172 in Acute Stroke Treatment”; vs., versus.

a Number (%).

b Median (IQR).

c Negative results imply a reduction of NIHSS or mRS in time course, positive results imply an increase of NIHSS or mRS in time course.

**Figure 3:**
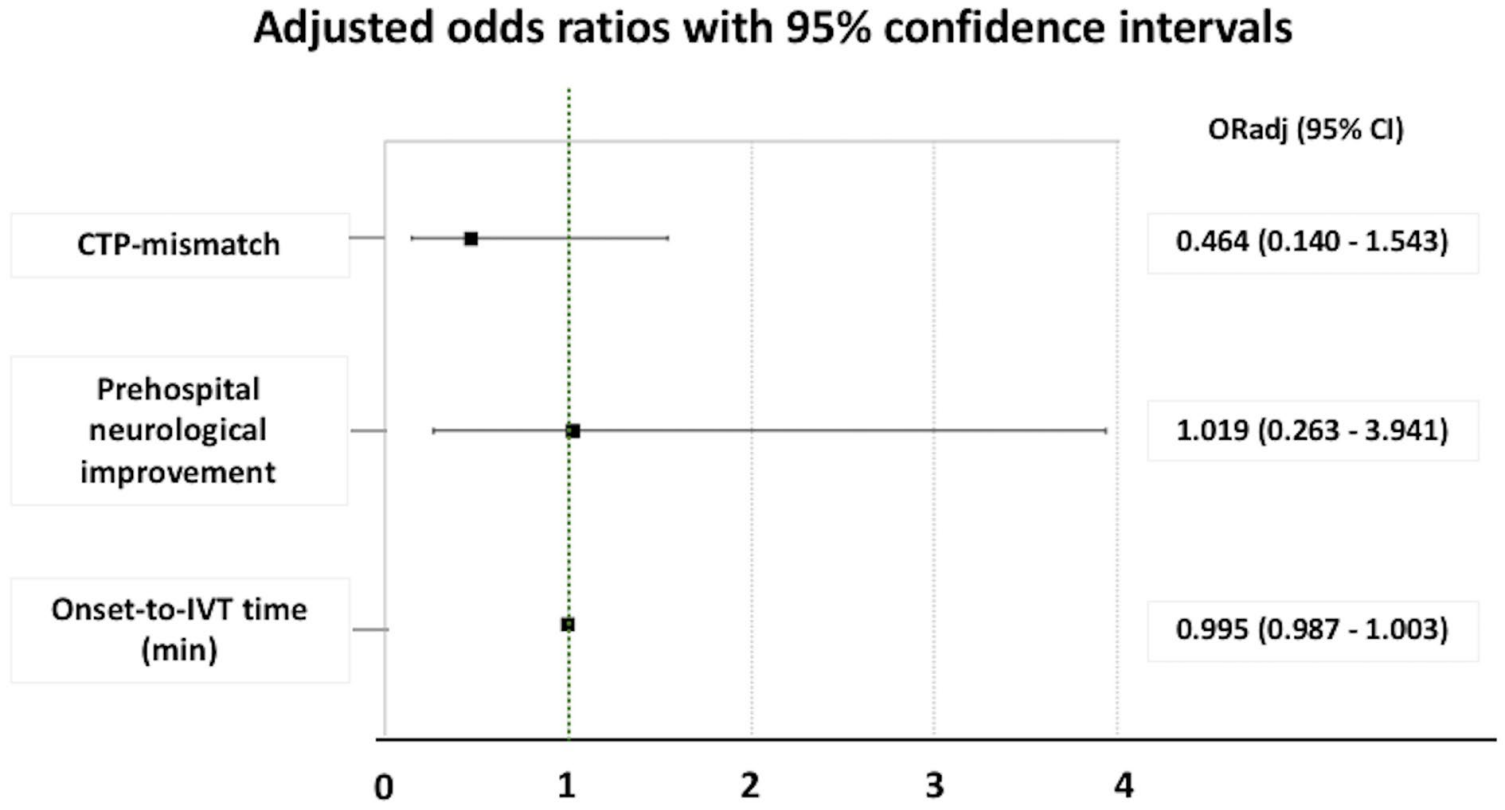
Adjusted odds ratio for good outcome (an mRS of 0–2) at 90 days in patients with minor ischemic stroke who underwent intravenous thrombolysis. OR_adj_, adjusted odds ratio; CI, confidence interval; CTP, computed tomography perfusion; IVT, intravenous thrombolysis.

Similarly, in logistic regression analyses after adjustment for baseline differences between CTP + IVT+ vs. CTP − IVT+ patients, no association between CTP mismatch and good functional outcomes at 90 days (defined as mRS 0–2) was disclosed (Figure 3).

#### 3.2.2 Prognostic yield of CTP mismatch on standard treatment outcomes in acute minor ischemic stroke patients (CTP + IVT− vs. CTP − IVT−)

Among patients without IVT, patients with CTP mismatch were found significantly more often with prehospital neurological improvement (72.2% vs. 37.8%, *p* = 0.01).

Hyperlipidemia was significantly more common in the CTP + IVT− group (57.9% vs. 27.7%, *p* = 0.02), with more frequent symptoms of hypesthesia (42.1% vs. 12.8%, *p* = 0.008) and aphasia (36.8% vs. 4.3%, *p* = 0.001) compared to CTP − IVT−. Microangiopathic stroke etiology (TOAST 3) was more frequent in CTP − IVT− (29.8% vs. 5.3%, *p* = 0.03).

Comparing outcome parameters of CTP + IVT− with CTP − IVT−, no significant differences in NIHSS after 24 h (*p* = 0.34), the NIHSS difference between admission and 24 h (*p* = 0.93), and the NIHSS difference between admission and discharge (*p* = 0.97) were disclosed. Similarly, there was no significant difference in the mRS difference between admission and discharge (*p* = 0.13) and in the mRS difference between admission and 90 days (*p* = 0.87) (Table 4).

## 4 Discussion

The present analysis comprised real-world data from a cohort of 267 minor ischemic stroke patients who were acutely admitted (within 4.5 h after stroke onset), with minor neurological deficits (NIHSS ≤5), and underwent multimodal CT imaging. The randomized PRISMS trial has previously demonstrated similar clinical outcomes in minor stroke patients with non-disabling neurological deficits who underwent IVT and those who underwent standard treatment (5). Our results yet indicate that in clinical practice, neurologists may be more prone to administer IVT to minor stroke patients in the presence of CTP mismatch (i.e., as reflected by the marginally higher probability of IVT administration in patients with vs. without CTP mismatch; *p* = 0.06). Based on the findings of the present analyses, we found no evidence in support of the utility of CTP for guiding decision-making regarding IVT or outcome prognostication in patients with minor ischemic stroke.

First, in the analysis of the whole cohort of minor ischemic stroke patients, CTP mismatch was not associated with good functional outcomes at 90 days. In addition, in sensitivity analysis (including only patients who underwent IVT), although IVT− related time-metrics were in favor of patients with CTP mismatch, i.e., shorter onset-to-admission time and shorter onset-to-thrombolysis time, patients without CTP mismatch performed significantly better in clinical outcomes, i.e., NIHSS difference between admission and 24 h and between admission and discharge. No significant differences in mRS after 90 days were uncovered when CTP + IVT+ patients were compared to CTP − IVT+, while mRS differences between admission and discharge and between admission and after 90 days were comparable between groups.

Second, among minor stroke patients who underwent standard treatment, no associations between CTP mismatch and clinical outcomes were disclosed. In particular, the comparisons between CTP + IVT− and CTP − IVT− patients revealed no significant differences in NIHSS after 24 h, NIHSS difference between admission and 24 h, and NIHSS difference between admission and discharge. Contrarily, lower mRS at discharge and at 90 days after the index event was noted in CTP + IVT− compared to CTP − IVT− patients but with similar mRS differences between admission and discharge and between admission and after 90 days between groups.

Taken together, the previous findings indicate that in minor ischemic stroke patients without LVO, CTP mismatch holds no prognostic relevance in predicting clinical outcomes in patients undergoing standard treatment nor may facilitate the selection of patients who mostly benefit from IVT. In addition to the established role of CTP mismatch in LVO, however, we should note that in clinical practice, CTP holds additional benefits for risk/benefit assessment, particularly in AIS cases with peripheral vessel occlusion, e.g., M3, because CT and CTA alone often cannot sufficiently capture the extent of ischemic tissue at risk. In previous studies, the combination of ncCT, CTA, and CTP was found to detect AIS with a sensitivity of 71% and specificity of 88% compared to ncCT alone (sensitivity 53% and specificity 84%) or combined ncCT with CTA (sensitivity 58% and specificity 85%) (23). In accordance with the previous evidence, it can be postulated that CTP mismatch may be a rather unreliable predictor of clinical benefit (i.e., with low sensitivity) but may be optimally used to exclude patients without mismatch from acute revascularization therapies (i.e., high specificity) (24). In line with this hypothesis, it is also noteworthy that in the present study, patients with CTP mismatch showed significantly more often prehospital neurological improvement (both the groups of patients who underwent IVT and standard treatment) than patients without CTP mismatch. This finding may indicate that hypoperfusion on CTP may “linger” while cerebral perfusion is being restored or that additional anatomical, pathophysiological, and technical reasons may account for persisting CTP mismatch despite clinical improvement (25).

Concerning the association between IVT and functional outcomes at 90 days, our results warrant caution in their interpretation. In contrast to the findings of the PRISMS trial (5), we found a significant association between IVT and good functional outcomes at 3 months following the index event. It should be stressed, however, that patients in the present study were not randomized or prospectively allocated to receive IVT or standard treatment; thus, inherent or unmitigable biases may exist (including CTP mismatch but also differences in baseline characteristics) that limit the generalizability of our findings. In addition, the two patient groups were unbalanced (75.3% received IVT vs. 24.7% received best medical treatment), while limitations associated with the retrospective study design may have further confounded our findings (e.g., the publication of the PRISMS trial results (5) during the study period).

Although in clinical practice, there is still equipoise regarding the off-label IVT use in patients with non-disabling neurological deficits and minor ischemic stroke (6); in the present cohort, IVT was overall safe with no occurrence of sICH according to ECASS-III criteria in IVT− treated patients. In accordance with our findings, sICH rates of approximately 2% have been reported in the relevant literature on IVT in acute minor ischemic stroke, as given in the studies by Sykora et al. (26), and Wang et al. (27). In addition, a meta-analysis by Lan et al. (28) including 10 original studies reported a risk of sICH of 3.8% in IVT− treated patients, but not all of the included studies applied the ECASS-III criteria. In line with these estimates, a case series of 73 minor stroke patients reported no sICH in IVT− treated or standard treatment patients, but 3 of 34 IVT− treated patients were found with asymptomatic ICH (14).

Regarding the underlying stroke etiology in the present cohort, it is not surprising that patients without CTP deficits presented more often with microangiopathic AIS. Differentiation of stroke etiology by CTP has been previously described in the literature. In the ASTRAL study, there was a positive association of cardioembolic stroke and a negative association of lacunar stroke with hypoperfusion on CTP (29). Similarly, another retrospective analysis of 182 AIS patients undergoing multimodal CT showed that microangiopathic stroke is more often “CTP negative,” whereas cardioembolic stroke is frequently accompanied by CTP deficit (30). Although in the present study, we could not detect a significant association of cardioembolic stroke with CTP mismatch, there was a trend toward higher prevalence of cardioembolic stroke in patients with vs. without CTP mismatch. In addition, data concerning the detection of lacunar stroke in CTP are inconsistent, with up to 50% of false negative results reported in CTP studies (31). Previous evidence indicates a sensitivity of 0 to 62.5% and specificity of 20% to 100% for the detection of lacunar stroke in CTP (31). In addition to technical parameters that may attenuate the reliability of CTP for lacunar stroke detection (31), heterogeneous pathophysiological mechanisms of subcortical infarcts may also account for the limited prognostic utility of CTP in this patient population (32).

### 4.1 Limitations

The results of the present study must be interpreted in light of certain methodological limitations. First, as this was an observational study in which IVT was administered as indicated by stroke neurologists, we cannot exclude that additional biases may have influenced the decision to administer or withhold IVT. Second, only a subset of clinically relevant baseline characteristics has been included in the present analyses; thus, our results warrant replication in prospective randomized controlled trials in which patient groups may be balanced for further confounders. Third, mRS and NIHSS often capture poorly deficits that may be “non-disabling” for the majority of AIS patients but may be perceived as “severely disabling” by others; therefore, the off-label use of IVT in such patients needs to be established in future studies. Fourth, additional parameters (such as collateral status) that may have influenced CTP mismatch were not included in the present analysis and should be addressed in future studies. Fifth, we cannot exclude that sample size constraints and further methodological limitations associated with the present study design may have accounted for type II errors; thus, larger prospective multicentric studies are required to corroborate these findings.

### 4.2 Conclusion

Even though IVT− treated patients with minor neurological deficits had favorable functional outcomes at 90 days compared to patients undergoing standard treatment, we found no evidence that CTP may aid the selection of minor stroke patients without LVO who may benefit from IVT. CTP mismatch also appeared to hold no prognostic relevance in predicting the clinical outcome of minor stroke patients who undergo standard treatment. Future randomized controlled clinical trials are direly needed to determine factors that could aid clinicians in deciding for or against IVT in selected patients with minor ischemic stroke presenting within the therapeutic time window.

## Data availability statement

The raw data supporting the conclusions of this article will be made available by the authors, without undue reservation.

## Ethics statement

The studies involving humans were approved by the Ethics Committee of the University of Tübingen (protocol number 189/2019BO2). The studies were conducted in accordance with the local legislation and institutional requirements. The ethics committee/institutional review board waived the requirement of written informed consent for participation from the participants or the participants’ legal guardians/next of kin because use of de-identified routine treatment data for research purposes is covered by clinic-wide consent.

## Author contributions

JS-P: Data curation, Formal analysis, Visualization, Writing – original draft, Writing – review & editing. ML: Data curation, Writing – review & editing. M-IS: Formal analysis, Validation, Visualization, Writing – original draft, Writing – review & editing. MK: Formal analysis, Writing – review & editing. FH: Data curation, Supervision, Writing – review & editing. UE: Data curation, Resources, Supervision, Writing – review & editing. SP: Conceptualization, Writing – review & editing. KF: Supervision, Writing – review & editing. UZ: Resources, Supervision, Validation, Writing – review & editing. AM: Conceptualization, Data curation, Formal analysis, Investigation, Methodology, Project administration, Supervision, Visualization, Writing – original draft, Writing – review & editing.
